# Diagnostic value of hematological parameters in the early diagnosis of acute cholecystitis

**DOI:** 10.1515/med-2025-1227

**Published:** 2025-07-24

**Authors:** Nedim Uzun, Ozgecan Gundogar, Naile Misirlioglu, Emine Yildirim, Neslin Sahin, Seyma Dumur, Hafize Uzun

**Affiliations:** Emergency Department, University of Health Sciences Hamidiye Faculty of Medicine, İstanbul, 34250, Turkey; Department of Pathology, Gaziosmanpasa Training and Research Hospital, İstanbul, 34250, Turkey; Department of Biochemistry, Gaziosmanpasa Training and Research Hospital, İstanbul, 34250, Turkey; Department of General Surgery, Gaziosmanpasa Training and Research Hospital, Istanbul, 34250, Turkey; Department of Radiology, Gaziosmanpasa Training and Research Hospital, Istanbul, 34250, Turkey; Department of Medical Biochemistry, Istanbul Atlas University, Istanbul, 34550, Turkey

**Keywords:** acute cholecystitis, diagnosis, neutrophils, systemic immune-inflammation index, systemic inflammatory response index

## Abstract

**Objectives:**

Accurate diagnosis of acute cholecystitis (AC) is critical because early laparoscopic cholecystectomy significantly reduces complications and mortality. This study evaluates the predictive value of inflammatory indices and hematological markers in diagnosing AC.

**Methods:**

A retrospective review was performed on early laparoscopic cholecystectomy cases at the Gaziosmanpaşa Training and Research Hospital in Istanbul between August 2013 and August 2023. Patient demographics, preoperative laboratory values, inflammatory indices – including neutrophil-to-lymphocyte ratio (NLR), systemic immune-inflammation index (SII), systemic inflammation response index (SIRI), and aggregate index of systemic inflammation (AISI) – were evaluated, along with hospital length of stay and histopathological outcomes.

**Results:**

Among 249 patients, 34 (13.6%) were diagnosed with AC, comprising 76 males (30.5%) and 173 females (69.5%), with a mean age of 48.9 ± 14.6 years. The median hospital length of stay was 3 days (range: 1–21). Significant elevations in both the SIRI and neutrophil count were observed in AC cases compared to controls (*P* < 0.001). ROC (receiver operating characteristic) curve analysis demonstrated comparable diagnostic performance for the SIRI (AUC = 0.746; 95% CI: 0.658–0.835; optimal cutoff: 1.98) and neutrophil count (AUC = 0.746; 95% CI: 0.658–0.835; optimal cutoff: 7.1 × 10^3^/μL) in predicting AC.

**Conclusions:**

The SIRI and neutrophil count are reliable markers that can improve the diagnostic accuracy and guide early management of AC.

## 1 Introduction

Acute cholecystitis (AC) predominantly results from gallstone-induced obstruction of the cystic duct or gallbladder neck, a pathology affecting 10–15% of the adult population worldwide [1]. While only 30% of the gallstone carriers develop symptoms, AC represents 3–10% of the emergency department presentations for acute abdominal pain [2,3,4].

Early surgical intervention is critical for managing AC, with laparoscopic cholecystectomy recommended within the first 72 h of presentation [5,6]. This approach has been associated with improved patient satisfaction, reduced complication rates, shorter hospital stays, and decreased mortality [7].

The Tokyo Guidelines (TG18/TG13) advocate a comprehensive diagnostic approach integrating clinical signs, biochemical markers, and imaging characteristics for AC diagnosis [8]. However, studies evaluating the diagnostic efficacy of the TG13 criteria are limited [9,10]. The reported diagnostic accuracy ranges from 60.4 to 94.0%, depending on whether pathological samples are used as the gold standard [9,11].

Recent studies have investigated the utility of inflammatory indices such as the systemic immune-inflammation index (SII), neutrophil-to-lymphocyte ratio (NLR), aggregate index of systemic inflammation (AISI), and systemic inflammation response index (SIRI) in differentiating AC and predicting its severity [12,13,14,15,16,17,18,19]. However, most research has focused on evaluating the severity and complications of AC, with limited evidence regarding their role in pathological diagnosis.

Berhuni et al. reported that elevated SII values are significantly associated with complicated acute appendicitis, supporting their utility in early risk assessment [20]. In addition, Tiwari et al. demonstrated that both the SII and SIRI are independently associated with disease severity and the risk of acute kidney injury [21]. Furthermore, Aktimur et al. found that elevated NLR levels have diagnostic value in acute mesenteric ischemia, correlating with adverse clinical outcomes [22]. These findings highlight the broader applicability of inflammatory indices in the diagnosis and prognostic evaluation of acute abdominal emergencies.

Therefore, this study aims to evaluate the predictive value of inflammatory indices and conventional laboratory markers in the diagnosis of AC.

## 2 Materials and methods

### 2.1 Study design and setting

This retrospective cohort study included all consecutive patients who underwent emergency laparoscopic cholecystectomy at the Gaziosmanpaşa Training and Research Hospital in Istanbul between August 2013 and August 2023. The study was approved by the Institutional Review Board (Approval Date: June 2023, Reference No: 122) and conducted in accordance with the principles outlined in the Declaration of Helsinki [23].

### 2.2 Patient selection

**Figure 1:**
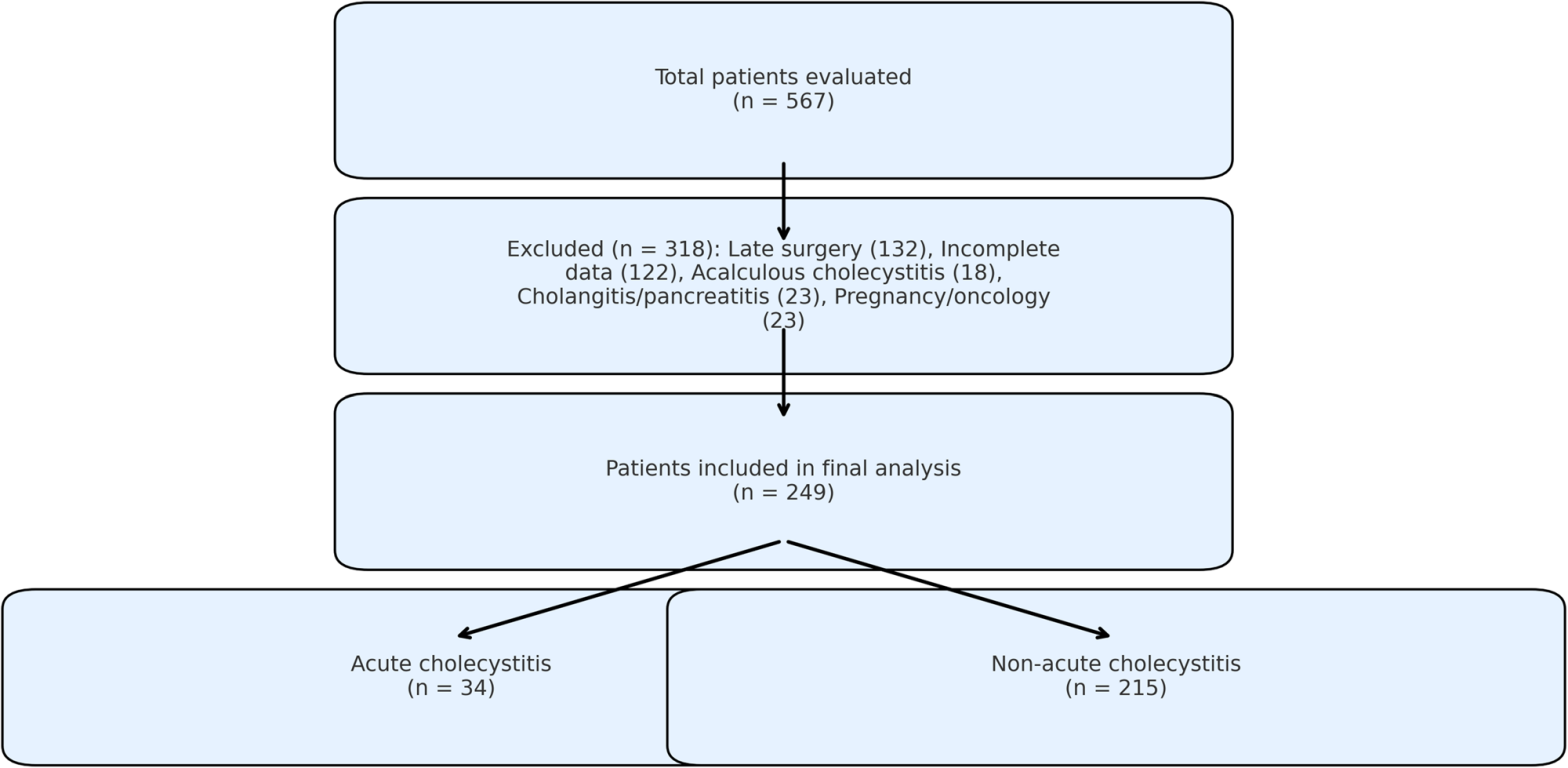
Flowchart of patient selection and grouping.

Eligible participants were defined as consecutive patients aged ≥17 years presenting with AC in the emergency department who subsequently underwent laparoscopic cholecystectomy within 72 h of admission. Exclusion criteria consisted of: (1) acalculous cholecystitis, (2) concurrent cholangitis or pancreatitis, (3) chronic inflammatory disorders known to affect acute-phase reactants (e.g., rheumatoid arthritis, inflammatory bowel disease), (4) current pregnancy or lactation status, and (5) active malignancy or recent chemotherapy exposure. The complete patient selection methodology, including enrollment flow and final group stratification based on histopathological confirmation of AC versus alternative diagnoses, is detailed in Figure 1.

### 2.3 Data collection and inflammatory indices

A comprehensive review of both electronic medical records and archived paper documentation was conducted. Systematic data extraction included demographic characteristics, admission laboratory values (complete blood count and conventional inflammatory markers), duration of hospitalization, and final histopathological reports.

The inflammatory indices were calculated as follows:

– NLR = neutrophils/lymphocytes.
– SII = (neutrophils × platelets)/lymphocytes.
– SIRI = (monocytes × platelets)/lymphocytes.
– AISI = (neutrophils × monocytes × platelets)/lymphocytes.

Final patient stratification was based on comprehensive histopathological examination of surgical specimens, with cases categorized as either AC (demonstrating characteristic neutrophilic infiltration, mural necrosis, and other diagnostic histological features) or non-AC (encompassing chronic cholecystitis with lymphocytic infiltration and fibrosis, or simple cholelithiasis without significant inflammatory changes).

### 2.4 Surgical procedure and surgeon experience

All procedures were performed using a standardized laparoscopic cholecystectomy protocol employing a four-port technique under general anesthesia. The operative technique systematically included: (1) careful dissection of Calot’s triangle with identification of critical anatomical structures, (2) double clipping and division of both the cystic duct and cystic artery, and (3) meticulous gallbladder dissection from the hepatic bed. Intraoperative decisions regarding peritoneal irrigation and closed suction drainage placement were made based on the presence of significant inflammation, bile spillage, or hemorrhage. All surgical interventions were conducted by or under the direct supervision of fellowship-trained general surgeons possessing board certification and a minimum of 5 years of specialized experience in advanced laparoscopic procedures. This standardized surgical approach was rigorously maintained throughout the study period to minimize technical variability and ensure consistent operative quality.

### 2.5 Statistical analysis

Statistical analyses were performed using SPSS software (version 18.0, SPSS Inc., Chicago, IL, USA). Normality of continuous variables was assessed using the Shapiro–Wilk test. Continuous data were presented as mean ± standard deviation (SD) or median with interquartile range (IQR), depending on the distribution. Categorical variables were summarized as frequencies and percentages. Between-group comparisons were conducted using independent samples *t*-tests or Mann–Whitney *U* tests for continuous variables and chi-square or Fisher’s exact tests for categorical variables. Correlations were analyzed using Spearman’s correlation coefficients.

Receiver operating characteristic (ROC) curves were used to evaluate the diagnostic accuracy of variables for AC. Optimal cutoff points were determined using Youden’s index and the maximum AUC. Sensitivity and specificity values were calculated for these cutoff points. A *P*-value of <0.05 was considered statistically significant.

## 3 Results

Out of the 567 patients evaluated in this study, 318 were excluded for the following reasons: failure to undergo surgery within the first 72 h (*n* = 132), incomplete data (*n* = 122), acalculous cholecystitis (*n* = 18), concomitant cholangitis or pancreatitis (*n* = 23), and cases involving pregnancy or oncology (*n* = 23). Consequently, 249 patients were included in the final analysis, of whom 76 (30.5%) were male and 173 (69.5%) were female. The mean patient age was 48.9 ± 14.6 years, and the median hospital length of stay was 3 days (range: 1–21 days).

Histopathological examination confirmed AC in 34 patients (13.6%), while the remaining 215 (86.4%) exhibited non-AC. The demographic, clinical, and laboratory characteristics of both groups are detailed in Table 1. Notably, gallbladder wall thickness and length of hospital stay were significantly greater in the AC group (*P* < 0.05). Furthermore, male sex was associated with an elevated risk of AC compared to females (relative risk [RR]: 1.96; 95% confidence interval [CI]: 1.33–2.89).

**Table 1:** Demographic and clinical characteristics of patients with and without AC

Variable	AC	Non-AC	*P*-value
	*N* = 34	*N* = 215	
**Demographics**			
Age (years)	50.5 (40.5–59.5)	48.0 (39.0–59.0)	0.716
Male, *n* (%)	18 (52.9%)	58 (27.0%)	**0.002**
**Clinical outcomes**			
Length of hospital stay (days)	3.98 (3.06–5.03)	2.59 (1.94–3.68)	**<0.001**
Pathological wall thickness (mm)	0.60 (0.40–0.70)	0.20 (0.20–0.30)	**<0.001**
**Laboratory parameters**			
Platelet count (10^9^/L)	246 (202–292)	238 (196–284)	0.778
MPV (fL)	9.1 (8.4–9.6)	9.3 (8.7–9.8)	0.430
WBC count (10^9^/L)	12.3 (10.6–14.2)	8.86 (7.22–11.2)	**<0.001**
Lymphocyte count (10^9^/L)	1.58 (1.10–2.15)	1.90 (1.40–2.54)	**0.014**
Monocyte count (10^9^/L)	0.59 (0.45–0.72)	0.49 (0.39–0.61)	**0.010**
Neutrophil count (10^9^/L)	9.91 (7.42–12.2)	5.92 (4.47–8.52)	**<0.001**
CRP (mg/L)	24.2 (10.3–94.6)	13.3 (6.47–28.1)	**0.003**
**Inflammatory indices**			
NLR	6.39 (3.91–10.41)	3.20 (1.83–6.01)	**<0.001**
SII	1607.15 (862.19–2331.06)	733.60 (430.20–1378.82)	**<0.001**
SIRI	3.78 (2.38–6.05)	1.55 (0.86–3.03)	**<0.001**
AISI	1610.12 (864.48–2335.18)	735.13 (431.18–1381.86)	**<0.001**

WBC (white blood cell count), CRP (C-reactive protein level), and MPV (mean platelet volume) are laboratory parameters. NLR (neutrophil-to-lymphocyte ratio), SII (systemic immune-inflammation index, calculated as platelet count × neutrophil count/lymphocyte count), SIRI (systemic inflammatory response index, calculated as neutrophil count × monocyte count/lymphocyte count), and AISI (aggregate inflammatory severity index) are inflammatory indices. Statistical significance was defined as *P* < 0.05. Data are presented as mean ± SD or median (25th–75th percentiles) as appropriate.

Significant differences in laboratory parameters between the AC and non-AC groups were observed for white blood cell (WBC) count, lymphocyte count, monocyte count, neutrophil count, and C-reactive protein (CRP) levels (*P* < 0.05). The SIRI, NLR, SII, and AISI were also significantly elevated in the AC group (*P* < 0.05).

**Figure 2:**
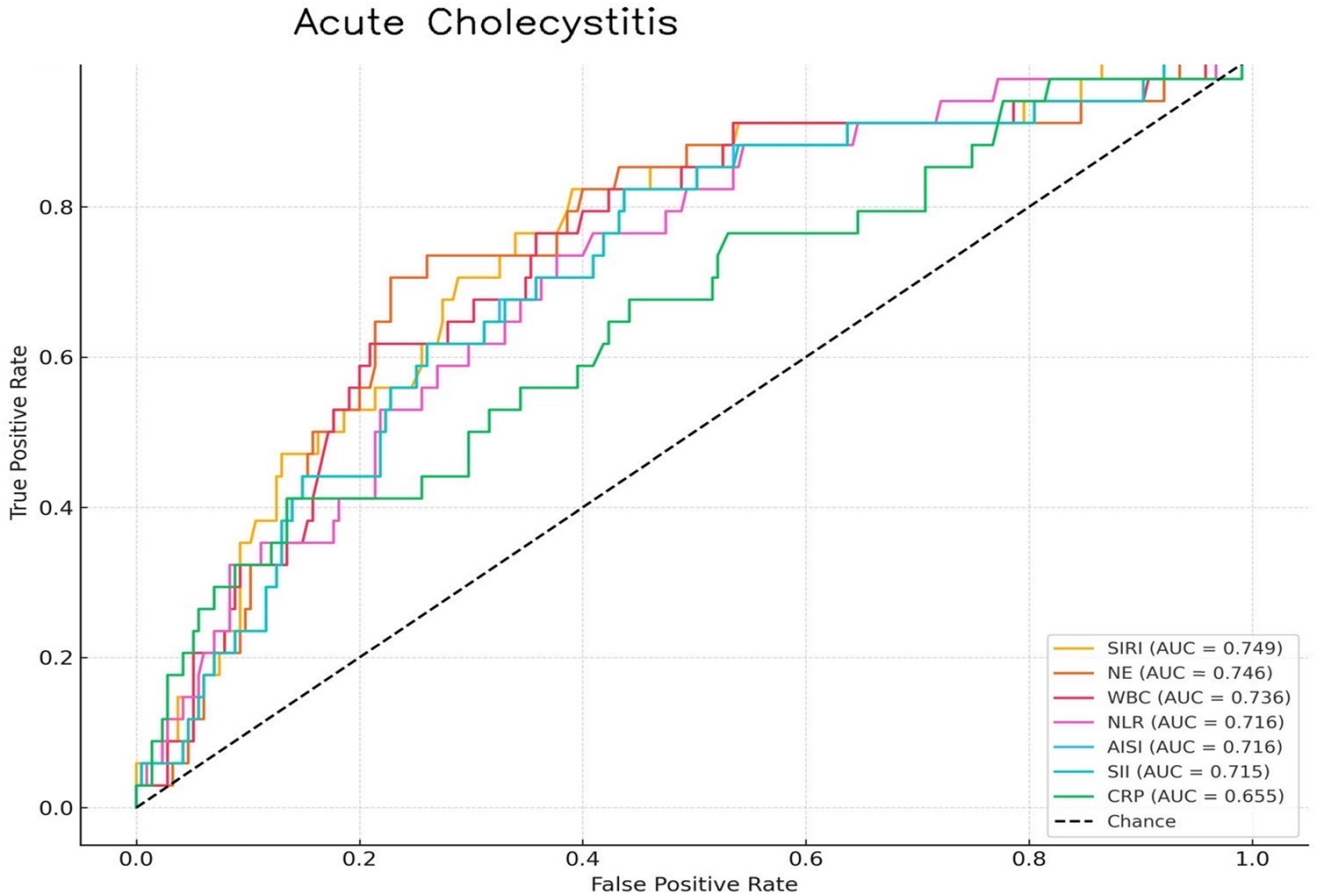
Receiver-operating characteristic curve analysis for predicting AC.

ROC curve analysis was conducted to evaluate the predictive accuracy of inflammatory indices and laboratory markers for AC (Table 2, Figure 2). SIRI showed the strongest predictive value among the indices, with an area under the curve (AUC) of 0.749 (95% CI: 0.663–0.834). At the optimal cutoff of 1.98, SIRI demonstrated 79% sensitivity and 61% specificity. Among laboratory markers, neutrophil count had the highest AUC value of 0.746 (95% CI: 0.658–0.835), with a cutoff value of 7.1 × 10^9^/L, yielding a sensitivity of 77% and a specificity of 63%.

**Table 2:** ROC curve analysis of hematologic parameters for AC

Parameter	AUC	SD	95% CI	Cutoff	Sensitivity (%)	Specificity (%)	*P*-value
SIRI	0.749	0.044	0.663–0.834	1.98	79	61	**<0.001**
NE	0.746	0.045	0.658–0.835	7.1	77	63	**<0.001**
WBC	0.736	0.006	0.723–0.748	9.7	80	60	**<0.001**
NLR	0.716	0.006	0.628–0.805	4.1	74	63	**<0.001**
AISI	0.716	0.046	0.626–0.805	963.9	71	64	**<0.001**
SII	0.715	0.046	0.625–0.805	964.8	71	65	**<0.001**
CRP	0.655	0.053	0.553–0.758	17.1	65	62	**<0.001**

AUC: area under the curve, SD: standard deviation, CI: confidence interval, WBC: white blood cell count, NE: neutrophil count, CRP: C-reactive protein, NLR: neutrophil-to-lymphocyte ratio, SII: systemic inflammatory index, SIRI: systemic inflammatory response index, and AISI: aggregate inflammatory systemic index. Significant values are presented in bold.

Neutrophil count exhibited strong correlations with WBC (*r* = 0.941; *P* < 0.001) and CRP (*r* = 0.507; *P* < 0.01). Additionally, significant correlations were observed between the inflammatory indices. Specifically, SIRI showed strong correlations with NLR (*r* = 0.905; *P* < 0.001), AISI (*r* = 0.877; *P* < 0.001), and SII (*r* = 0.876; *P* < 0.01).

## 4 Discussion

This study found that the SIRI and neutrophil count were significantly elevated in patients with AC compared to controls. ROC curve analysis identified optimal cutoff values of >1.98 for SIRI and >7.1 × 10^9^/L for neutrophil count, which effectively predicted AC with sensitivities of 79 and 77%, respectively.

Previous studies have reported that the sensitivity of WBC count for diagnosing gangrenous cholecystitis ranges from 60 to 77% [24,25]. Similarly, the diagnostic value of CRP has been highlighted, with sensitivities reported from 71 to 100% [12,26,27,28]. In contrast to previous studies relying on clinical and radiological criteria, the present study utilized histopathological evaluation as the gold standard for definitive diagnosis. Our results revealed that WBC count exhibited superior sensitivity in distinguishing AC from non-acute cases compared to previous reports.

In this study, consistent with the findings reported by Naidu et al., neutrophil count emerged as an independent predictor of AC, with close cutoff values observed in both studies [11]. The use of histopathology as the gold standard may explain the agreement in cutoff values.

Inflammatory indices, including SIRI, NLR, SII, and AISI, have been extensively investigated as predictors of AC severity or conversion to open cholecystectomy [12,13,14,15,16,17,18,19,20,21,22]. However, to our knowledge, this study evaluated the utility of SIRI and AISI in the pathological diagnosis of AC. Additionally, SIRI demonstrated strong correlations with other indices, such as NLR, SII, and AISI, reinforcing its potential reliability as a diagnostic marker.

In addition to AC, the diagnostic utility of hematologic markers has been explored in various abdominal emergencies. Yazar et al. reported that elevated NLR and PLR values could predict the need for surgical intervention in small bowel obstruction [29]. Similarly, in acute diverticulitis, Özdemir et al. observed significantly higher NLR levels in patients with complicated disease, highlighting its role in early risk stratification [30]. Furthermore, Şahin et al. demonstrated markedly elevated SII and SIRI levels in patients with incarcerated inguinal hernia, indicating potential tissue ischemia [31]. Collectively, these findings emphasize the broader applicability of systemic inflammatory indices in the early evaluation of acute surgical pathologies – a conclusion further supported by the present study.

This study had several limitations. The retrospective and single-center design may limit the generalizability of the findings to broader patient populations. Additionally, the study lacked comprehensive data on medical history, physical examination findings, and imaging results. The imbalance in group distributions and the exclusion of certain patients may have introduced selection bias. Furthermore, subgroup analysis within AC was not performed. Comparing subgroups could have enhanced the study’s clinical relevance. Despite these limitations, this study is one of the few investigations utilizing histopathology as the gold standard for diagnosing AC. Future multicenter prospective studies are needed to validate these findings.

## 5 Conclusions

In conclusion, this study identified the SIRI and neutrophil count as valuable predictors of AC in emergency department settings. The SIRI >1.98 and neutrophil count >7.1 × 10^9^/L could serve as reliable markers for the management of suspected AC cases. Large-scale prospective multicenter studies are warranted to validate these findings and enhance the diagnostic accuracy for AC.
